# The impact of far-UVC radiation (200–230 nm) on pathogens, cells, skin, and eyes – a collection and analysis of a hundred years of data 

**DOI:** 10.3205/dgkh000378

**Published:** 2021-02-16

**Authors:** Martin Hessling, Robin Haag, Nicole Sieber, Petra Vatter

**Affiliations:** 1 Institute of Medical Engineering and Mechatronics, Ulm University of Applied Sciences, Ulm, Germany

**Keywords:** radiation disinfection, far-UVC, excimer lamp, 222 nm, coronavirus, influenza virus

## Abstract

**Background:** The ongoing coronavirus pandemic requires new disinfection approaches, especially for airborne viruses. The 254 nm emission of low-pressure vacuum lamps is known for its antimicrobial effect, but unfortunately, this radiation is also harmful to human cells. Some researchers published reports that short-wavelength ultraviolet light in the spectral region of 200–230 nm (far-UVC) should inactivate pathogens without harming human cells, which might be very helpful in many applications.

**Methods:** A literature search on the impact of far-UVC radiation on pathogens, cells, skin and eyes was performed and median log-reduction doses for different pathogens and wavelengths were calculated. Observed damage to cells, skin and eyes was collected and presented in standardized form.

**Results:** More than 100 papers on far-UVC disinfection, published within the last 100 years, were found. Far-UVC radiation, especially the 222 nm emission of KrCl excimer lamps, exhibits strong antimicrobial properties. The average necessary log-reduction doses are 1.3 times higher than with 254 nm irradiation. A dose of 100 mJ/cm^2^ reduces all pathogens by several orders of magnitude without harming human cells, if optical filters block emissions above 230 nm.

**Conclusion:** The approach is very promising, especially for temporary applications, but the data is still sparse. Investigations with high far-UVC doses over a longer period of time have not yet been carried out, and there is no positive study on the impact of this radiation on human eyes. Additionally, far-UVC sources are unavailable in larger quantities. Therefore, this is not a short-term solution for the current pandemic, but may be suitable for future technological approaches for decontamination in rooms in the presence of people or for antisepsis.

## Zusammenfassung

**Hintergrund:** Die anhaltende Coronavirus-Pandemie erfordert neue Desinfektionsansätze, besonders für Viren in der Luft. Die 254 nm Emission von Niederdruck-Quecksilberdampflampen ist bekannt für ihre antibakterielle Wirkung, allerdings ist diese Art der Bestrahlung auch für menschliche Zellen schädlich. Einige Forscher veröffentlichten Berichte, dass kurzwelliges ultraviolettes Licht im Spektralbereich von 200–230 nm (Far-UVC) Krankheitserreger inaktiviert, ohne dabei menschlichen Zellen zu schaden, was für viele Anwendungen sehr hilfreich sein könnte.

**Methoden:** Es wurde eine Literaturrecherche zum Einfluss von Far-UVC-Strahlung auf Krankheitserreger, Zellen, Haut und Augen durchgeführt und die log-Reduktionsdosen für verschiedene Krankheitserreger und Wellenlängen berechnet. Beobachtete Schäden an Zellen, Haut und Augen wurden gesammelt und in standardisierter Form dargestellt.

**Ergebnisse:** Insgesamt wurden mehr als 100 Arbeiten zur Far-UVC-Desinfektion gefunden, die in den letzten ungefähr 100 Jahren veröffentlicht wurden. Besonders 222 nm Emissionen von KrCl-Excimer-Lampen weisen starke antimikrobielle Eigenschaften auf. Die durchschnittlich benötigten log-Reduktionsdosen sind um den Faktor 1,3 höher als bei einer 254 nm Bestrahlung. Eine Dosis von 100 mJ/cm² reduziert alle Krankheitserreger um mehrere Größenordnungen, ohne dabei menschliche Zellen zu zerstören, wenn langwellige Emissionen über 230 nm durch optische Filter blockiert werden.

**Schlussfolgerung:** Der Ansatz ist sehr vielversprechend, speziell was den zeitlich begrenzten Einsatz angeht, jedoch sind die hierzu verfügbaren Daten relativ spärlich. Untersuchungen mit hohen Far-UVC-Dosen über einen längeren Zeitraum wurden noch nicht durchgeführt und es gibt noch keine positiven Studien über den Einfluss dieser Strahlung auf das menschliche Auge. Zudem sind Far-UVC-Quellen nicht in größeren Mengen verfügbar. Daher stellt das keine kurzfristige Lösung für die aktuelle Pandemie dar, aber ist vielleicht geeignet für künftige technologische Lösungen zur Dekontamination in Räumen in Anwesenheit von Menschen oder zur Antiseptik.

## Introduction

The ongoing severe acute coronavirus (SARS-CoV-2) pandemic is currently leading to an intensified worldwide search for approaches to inactivate viruses and other pathogens, especially in the air. The antimicrobial properties of ultraviolet radiation from mercury vapor lamps are well known and have been applied for over a hundred years [1]. They have also proven to be effective against coronaviruses [2], [3].

Mercury vapor lamps exhibit an emission peak at 254 nm in the ultraviolet spectral range, known as UVC, which extends from 200 to 280 nm. This radiation is absorbed by deoxyribonucleic acid (DNA) and ribonucleic acid (RNA) and leads to DNA and RNA damage, e.g., the formation of pyrimidine dimers [4], [5], [6].

Unfortunately, this radiation is also harmful to human cells and tissue, which also contain DNA. The possible consequences of skin irradiation include erythema formation and potentially carcinogenic mutations, while photokeratitis is among the potential eye lesions [7], [8], [9]. 

In 2004, Sosnin et al. [10] published an investigation on the impact of short-wavelength (206 nm) UVC light on Chinese hamster ovary (CHO) cells and with 222 nm irradiation of *Escherichia coli* (*E. coli*). While the *E. coli* were reduced by more than 3 orders of magnitude using a dose of 100 mJ/cm^2^, there was no detectable damage to the CHO cells for doses up to 400 mJ/cm^2^. Consequently, Sosnin et al. suggested applying this short-wavelength UV light for wound decontamination to prevent surgical site infections. This idea was examined more closely by Buonanno et al. [11], [12]. They also coined the term “far-UVC” for short-wavelength UVC light in the range between approximately 200 and 230 nm. In subsequent publications, Welch et al. and Buonanno et al. demonstrate that this far-UVC light seems suitable for the inactivation of influenza and coronaviruses in the air at doses that do not damage human cells [13], [14]. 

**Figure 1:**
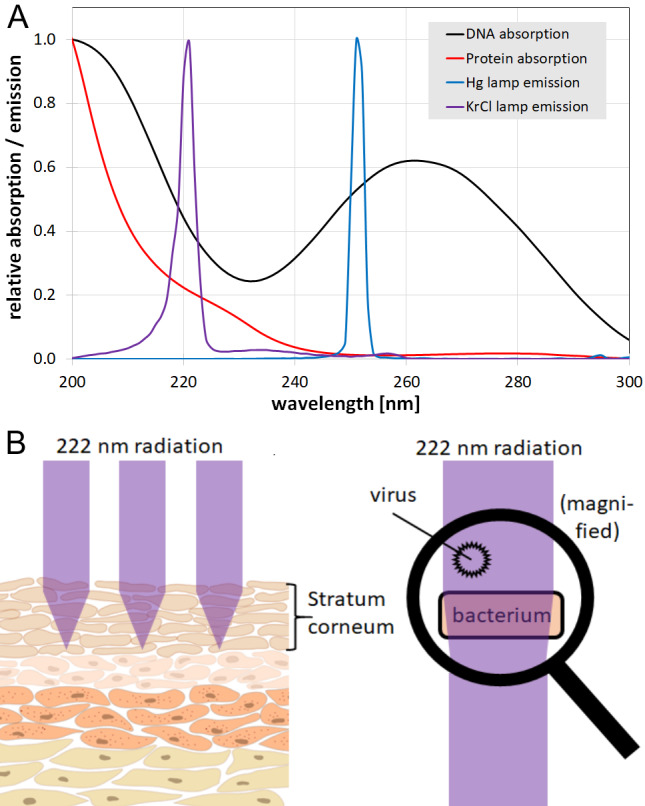
A: Absorption spectra of DNA and proteins, with emission spectra of a KrCl excimer and a low-pressure mercury lamp (Hg lamp); B: Scheme of far-UVC propagation in skin and pathogens

This advantageous characteristic of far-UVC radiation is probably due to the strong protein absorption (Figure 1A (Fig. 1)) and the larger size of human cells compared to most microorganisms. While bacteria or viruses (typical diameter 1 µm and 0.1 µm, respectively) are irradiated completely and without much attenuation, less than 5% of far-UVC radiation reaches the center of a mammalian cell with a typical diameter of more than 10 µm [15]. Human skin is assumed to be further protected against far-UVC radiation by the stratum corneum, the outermost layer of the epidermis, consisting of dead keratinocytes that absorb most of the ultraviolet radiation (Figure 1B (Fig. 1)) [11], [16], [17]. Additionally, for the eye, it is supposed that it is the cornea and its tear layer that protects the lens by absorbing far-UVC radiation [11], [13], [18].

The promising reports on the effects of far-UVC on cells, tissue and pathogens – including coronaviruses – give reason to hope that this radiation might become a very important tool in the fight against airborne pathogens and especially SARS-CoV-2 in the current pandemic. However, there still seems to be only a very limited number of animal or human studies; among them, there exists at least one investigation describing the formation of erythema and cyclobutane pyrimidine dimers (CPD) after 222 nm irradiation [19].

The aim of this study was to collect and analyze the results published to date on the impact of far-UVC in the spectral region between approximately 200 and 230 nm on pathogens, animal and human cells, skin and eyes, as well as to find further information regarding the safety of this kind of radiation and acquire data to determine the necessary doses for pathogen reductions.

## Materials and methods

A search was performed in PubMed and Google Scholar using various combinations of the following terms:

- “far-UVC”,
- “deep UV”,
- “excimer lamp”,
- “207 nm”,
- “211 nm”,
- “222 nm”,
- “230 nm”,
- “disinfection”,
- “inactivation”,
- “photoinactivation”,
- “action spectrum”,
- “cells”,
- “skin”, and
- “eye”.

References in the retrieved literature were examined for their possible inclusion in this study. References citing the identified literature were also checked.

The results were divided into microorganisms (including bacteria, bacterial spores, fungi, viruses, and protists), human and animal cells, skin and eye. If results on microorganism inactivation for different irradiation doses were published in one report, those describing a reduction by approx. 3 log levels were selected, and the necessary dose for a 90% (1 log-)reduction was calculated. Results presented only as figures without exact values in the text or tables were read from enlarged figures. Combinations of different inactivation techniques were ignored, as were different reactivation approaches after irradiation. Only experiments with irradiation wavelengths between 200 and 235 nm were included in the analysis and divided into sections termed “210 nm” (200–215 nm), “222 nm” (216–225 nm) and “230 nm” (226–235 nm) for simplification. 

For comparison with the effect of mercury vapor lamp emissions, a fourth section termed “254 nm” (248–259 nm) was included in the data collection, but no extensive literature search was performed for this wavelength. In case the far-UVC study was not accompanied by 254 nm results, other literature values were consulted. If there were multiple results for one microorganism, the median values were calculated to reduce the influence of outliers caused by stronger absorbing media or mutants with higher or lower UVC sensitivity.

## Results

### Inactivation of Microorganisms 

More than 100 studies on the impact of far-UVC radiation on microorganisms were found in the literature. Among them, many investigations dated from the first half of the last century and some were even performed about a hundred years ago, an impressive accomplishment considering the available (lamp) technology at that time. Unfortunately, not all of these investigations could be included in the following analysis because the inactivated microorganisms were not determined, or the inactivation doses were given in dimensions such as energy per volume, which could not be converted to today’s typical dose dimension of energy per area [20], [21], [22], [23], [24], [25]. The more recent far-UVC investigations were mostly performed with krypton chloride (KrCl) excimer lamps with a peak emission at 222 nm. Only a few researchers applied a broadband UVC source in combination with a monochromator or optical filters or even lasers.

The data collection – about 250 single far-UVC results for 14 bacterial species, 9 bacterial spores, 5 fungi, 23 viruses and one protist – can be found in Table 1 (Tab. 1), divided into bacteria, bacterial spores, fungi, viruses, and protists and sorted by microorganism, wavelength and media (L: liquid, S: surface, A: air/aerosol), which were in most cases liquids (salt solutions). For each microorganism (species), the median value for the log-reduction dose was determined and compared to the median log-reduction dose for the wavelength of 254 nm of mercury vapor lamps by calculation of the log-reduction dose ratio.

### Impact of far-UVC on human and animal cells, skin and eye

For the impact on human and animal cells, skin and eye, only 15 publications could be retrieved. Most of them are of recent origin, but one impressive study was performed almost 90 years ago.

The results are listed in Table 2 (Tab. 2), which is divided into experiments involving “cells”, “skin” and “eyes”. Unfortunately, the comparison of the different results is complicated by the authors’ investigation of different possible observable phenomena, such as cell number/viability, epidermal thickening, dimer formation and erythema formation. Therefore, not all examined parameters are listed in Table 2 (Tab. 2) but only the most frequently mentioned ones, e.g., cyclobutane pyrimidine dimer (CPD) formation. 

## Discussion

### Inactivation of Microorganisms

There are large variations in the necessary far-UVC log-reduction dose between different microorganisms and sometimes even between different strains of one species, but in all reports, far-UVC is a very powerful antimicrobial radiation. 

Additional differences can be found for different media. The observed reduction doses of pathogens in the air/aerosols are very low (about 1 mJ/cm^2^), but unfortunately this is based on only two investigations on human coronaviruses and influenza virus. Most results are available for microorganisms in liquids and in about 10 cases on surfaces, with much higher necessary irradiation doses for these surfaces. Most of these surfaces were not totally smooth, but exhibited pores, such as different natural skins or agar, which may have provided a kind of shade against the far-UVC irradiation.

Nevertheless, for 2/3 of bacteria, bacterial spores, fungi, viruses, and protists, a dose of 10 mJ/cm^2^ is sufficient for a 90% or higher reduction for all media. With a dose of 100 mJ/cm^2^, almost all examined pathogens are inactivated by several orders of magnitude.

The antimicrobial property of 254-nm irradiation is assumed to be mostly based on DNA or RNA damage [4], [26], [27], [28], [29], [30], [31], [32]. However, if the far-UVC inactivation mechanism were caused by DNA/RNA damage alone, far-UVC should be much less effective than 254 nm radiation, because of the lower DNA and higher protein absorption at shorter wavelengths (see Figure 1 (Fig. 1)) and the lower number of incident photons per mJ at this wavelength compared to 254 nm. 

In fact, higher average necessary log-reduction doses in liquids were observed for all far-UVC wavelengths. The calculated required increase is 1.8, 1.3, and 3.3 times higher for 210 nm, 222 nm, and 230 nm, respectively, compared to the 254 nm log-reduction doses. These values are not very precise, especially those for 210 nm and 230 nm, because of the very limited available data. Nevertheless, 222 nm irradiation seems to be more effective than 210 and 230 nm.

The more comprehensive 222 nm results in liquids even allow to distinguish between the log-reduction doses necessary for the different types of pathogens: bacteria x1.2, bacterial spores x0.7, fungi x1.1 and viruses x1.7. Thus, 222 nm irradiation seems to be especially suited for spore inactivation.

Deviations from the expected damage caused only by DNA destruction are suspected to have their origin in the additional protein absorption and lethal protein damage [11], [33], [34], [35]. 

### Impact of far-UVC on human and animal cells, skin and eye

The investigations listed in Table 2 (Tab. 2) are not very numerous and were performed on different kinds of research objects (cells, skin, eyes) and analyzed for different parameters (CPD, erythema, cell survival and other kinds of damage), which makes comparison difficult. However, at least for skin, there is a rather complete data set on CPD formation and some complementary results on erythema; furthermore, the corresponding irradiation doses are available for all lesions that occurred.

The majority of the presented studies conclude that human and animal cells can tolerate far-UVC doses of 150 mJ/cm^2^ for 207 nm irradiation – and probably even much higher ones for 222 nm – without damage such as dimer formation, erythema or increased cell death. This irradiation dose is much higher than 1.7 mJ/cm^2^, the only 222 nm dose published to date for a 3 log-reduction of coronaviruses in aerosols, and still many times above the previously mentioned 10 mJ/cm^2^ for a one log-reduction of 2/3 of the pathogens in Table 2 (Tab. 2). In fact, even 100 mJ/cm^2^, the dose necessary to inactivate all listed microorganisms by several orders of magnitude, seems to be harmless according to the majority of investigations.

Unfortunately, these investigations stand in contrast to at least five studies in which cell lesions were observed at much lower doses [19], [36], [37], [38], [39], in two of these even for doses below 1 mJ/cm^2^ [36], [37].

One reason for this apparent contradiction regarding the experiments on chicken embryo cells, Chinese hamster ovary cells and grasshopper neuroblasts might be the different cell types and the lack of protection by the stratum corneum or tear layer. For the study by Woods et al. – one of only three studies on real human skin – it is speculated that the observed CPD and erythema formation at low doses might be caused by the longer UV wavelength emission of the KrCl excimer lamp. In contrast to many other setups, Wood et al. employed no optical filter to block emissions of longer UVC wavelengths. As can be concluded from Table 2 (Tab. 2), even low doses of lamp emissions around 254 nm would lead to cell lesions. This assumption of negative effects of longer wavelength emissions is supported by the results of Yamano et al. [40], who worked with different long-wavelength cut-off filters and observed better results (no lesions even at very high far-UVC doses) with improved filters. Unfortunately, the exact radiation spectra for Pitts’ eye experiments are unknown; therefore it cannot be clarified whether their light source also emitted at longer wavelengths, which would have solved the last contradiction. 

It is noticeable that most positive studies were conducted with the participation or support of Ushio, the leading manufacturer of far-UVC lamps, which might raise suspicions. In fact, the involvement of Ushio has probably influenced the results, but only by providing advanced lamp and filter techniques, as can be seen in the study by Yamano et al., [40] with two different Ushio lamp prototypes which had different filter properties concerning transmission above 250 nm.

However, even if the results of these 5 studies could be invalidated by cell type, protection and long-wavelength emissions, the fact remains that the total number of successful studies is quite small to date. Studies on the impact of 222 nm irradiation on the human eye have not yet been carried out at all, and among the skin investigations, only two positive ones have been performed on humans. One of these used very high doses of up to 18,000 mJ/cm^2^ [41], but only on a single person. A daily exposure to far UVC radiation for several years might result in much higher total doses. Another aspect that has not been investigated yet is the potential impact of a repeated far-UVC skin irradiation on the skin’s microbiome, which might shift towards more far-UVC resistant microorganisms.

## Conclusions

Far-UVC – especially at a wavelength of 222 nm – is very effective against all pathogens. The average necessary log-reduction doses are slightly higher compared to UVC irradiation with a 254 nm low-pressure mercury vapor lamp. A dose of 100 mJ/cm^2^ should reduce most pathogens in most media by several orders of magnitude without harming human skin or eyes. Therefore, the prevention of surgical site infections by far-UVC irradiation – as suggested by Sosnin et al. [10] and Buonanno et al. [11] – seems to be very plausible and attractive.

Far-UVC also raises hopes in the fight against viruses, as suggested by Welch et al. [13] and Buonanno et al. [14], but it will probably not offer a short-term, large-scale solution for two reasons:

1. The safety of far-UVC irradiation is not yet guaranteed, despite the predominantly very positive results of the last years. Even if the few observed lesions are due to avoidable long-wavelength UVC emissions, even the successful studies were only carried out with limited doses and durations and only in two successful studies on humans. These do not yet exclude possible damage in applications over long periods of time (months to years) with even higher total doses.
2. Mercury vapor lamps are readily available worldwide in all wattages. This does not apply to far-UVC sources. Far-UVC LEDs currently have outputs in the mW range and lifetimes of hundreds of hours. This means that suitably powerful LED light sources are still years away. Excimer lamps, which were also used in most of the studies presented, are much more highly developed and have lifetimes of 10,000–100,000 hours. However, they are only commercially manufactured by a handful of companies worldwide and are only available in very limited quantities.

Therefore, this is not a short-term solution for the current pandemic, but may be suitable for future technological approaches for decontamination in rooms in the presence of people or for antisepsis. 

## Notes

### Competing interests

The authors declare that they have no competing interests.

### Funding

The authors did not receive any funds.

## Figures and Tables

**Table 1:**
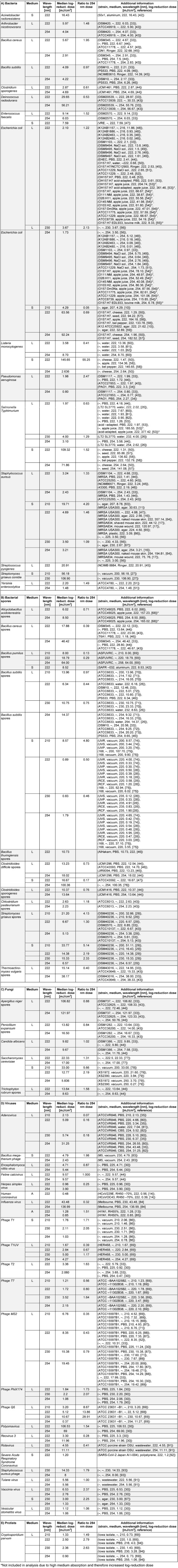
Far-UVC inactivation data for different microorganisms and wavelengths: A) bacteria, B) bacterial spores, C) fungi, D) viruses and E) protists; (L: liquid, S: surface, A: air/aerosol)

**Table 2:**
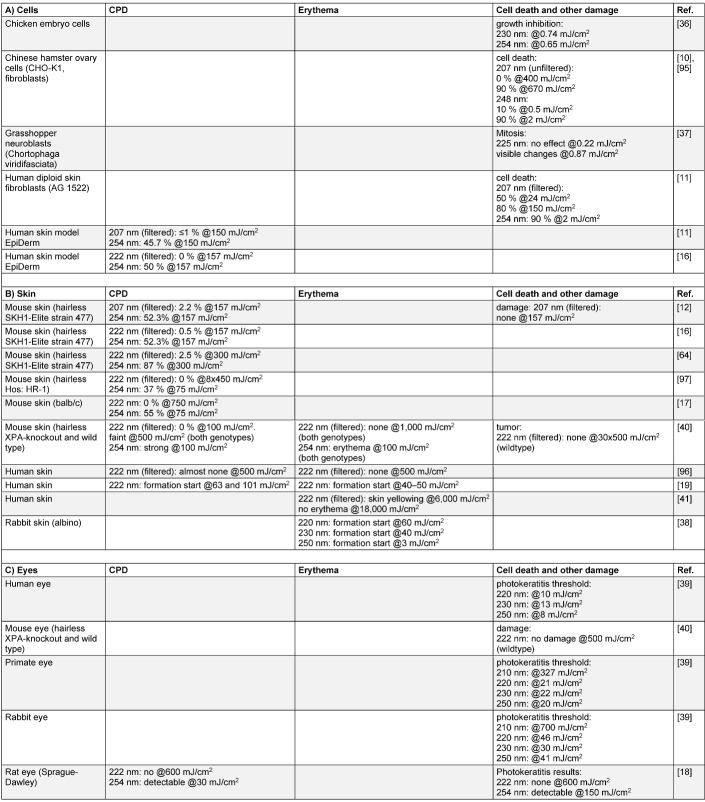
Impact of far-UVC on human and animal cells (A), skin (B), and eye (C)
